# Ligand-Based Pharmacophore Modeling, Molecular Docking, and Molecular Dynamic Studies of Dual Tyrosine Kinase Inhibitor of EGFR and VEGFR2

**DOI:** 10.3390/ijms21207779

**Published:** 2020-10-21

**Authors:** Frangky Sangande, Elin Julianti, Daryono Hadi Tjahjono

**Affiliations:** 1 School of Pharmacy, Bandung Institute of Technology, Jalan Ganesha 10, Bandung 40132, Indonesia; frangky.sangande@gmail.com (F.S.); elin_julianti@fa.itb.ac.id (E.J.); 2 Study Program of Pharmacy, Faculty of Science and Technology, Universitas Prisma, Jalan Pomorow 113, Manado 95126, Indonesia

**Keywords:** dual inhibitor, EGFR, VEGFR2, ligand-based pharmacophore, molecular docking, molecular dynamics

## Abstract

Epidermal growth factor receptor (EGFR) and vascular endothelial growth factor receptor 2 (VEGFR2) play an important role in cancer growth. Both of them have close relationships. Expression of EGFR will induce an angiogenic factor (VEGF) release for binding with VEGFR2. However, the existence of VEGF up-regulation independent of EGFR leads to cancer cell resistance to anti-EGFR. Therefore, a therapeutic approach targeting EGFR and VEGFR2 simultaneously may improve the outcome of cancer treatment. The present study was designed to identify potential compounds as a dual inhibitor of EGFR and VEGFR2 by the computational method. Firstly, the ligand-based pharmacophore model for each target was setup to screen of ZINC database of purchasable compounds. The hit compounds obtained by pharmacophore screening were then further screened by molecular docking studies. Taking erlotinib (EGFR inhibitor) and axitinib (VEGFR2 inhibitor) as reference drugs, six potential compounds (ZINC08398597, ZINC12047553, ZINC16525481, ZINC17418102, ZINC21942954, and ZINC38484632) were selected based on their docking scores and binding interaction. However, molecular dynamics simulations demonstrated that only ZINC16525481 and ZINC38484632 which have good binding free energy and stable hydrogen bonding interactions with EGFR and VEGFR2. The result represents a promising starting point for developing potent dual tyrosine kinases inhibitor of EGFR and VEGFR2.

## 1. Introduction

Tyrosine kinases are enzymes that catalyze the phosphorylation of tyrosine residues in proteins and activate signal transduction pathways that play an important role in cell proliferation, differentiation, migration, metabolism, and apoptosis. The existence of mutation and overexpression in these enzymes will trigger cancer formation [1]. Epidermal growth factor receptor (EGFR) is a member of tyrosine kinases and is commonly overexpressed in some types of cancer, such as non-small-cell lung cancer, breast, esophageal, cervical, and head and neck cancer [2]. The inhibition of EGFR activity is a rational strategy in design of anticancer [3]. There are two main groups of EGFR inhibitors in clinical use, i.e., monoclonal antibodies (mAbs) and small-molecule tyrosine kinase inhibitors (TKIs). mAbs compete with the endogenous ligand for receptor binding in the extracellular domain of EGFR, while TKIs work by competing with ATP in the intracellular catalytic domain, thereby preventing enzyme activity [4].

In pathologic conditions, EGFR activation will increase the expression of vascular endothelial growth factor (VEGF). When VEGF binds to vascular endothelial growth factor receptor (VEGFR), especially VEGFR2, the process of angiogenesis will begin [5]. VEGFR is also a member of the tyrosine kinase group that demonstrates a role in cancer growth through the angiogenesis mechanism, therefore it is an ideal drug target in cancer therapy. However, there are several mechanisms of expression of VEGF independent of EGFR. Overexpression of VEGF by this pathway is associated with cancer cell resistance to anti-EGFR [6]. Therefore, therapeutic approaches using drugs that inhibit both EGFR and VEGFR2 can increase the efficiency of cancer therapy and overcome the resistance problem [7,8]. In 2011, the US Food and Drug Administration (US FDA) approved the use of vandetanib, targeting EGFR and VEGFR2, for the treatment of medullary thyroid cancer [9]. The inhibition activity of vandetanib is based on the 4-anilino-quinazoline scaffold. Several studies have tried to design and develop new compounds as dual tyrosine kinases inhibitor of EGFR and VEGFR2 by generating the 4-anilino-quinazoline derivatives such as 2-chloro-4-anilino-quinazoline [10], 4-anilino-quinazoline-urea [11]. In other studies, benzimidazole [12] and phthalazine derivatives [13] have been tested for their activity against EGFR and VEGFR2.

In drug discovery, computational methods have been applied widely for identifying new drug candidates against the individual target. In particular, screening of large virtual libraries by molecular docking, pharmacophore modeling, quantitative structure-activity relationship (QSAR), and combination methods have been used for lead discovery of EGFR [14,15] and VEGFR2 inhibitor [16,17] as an individual target. However, very few computational methods were employed for identifying chemical compounds that can inhibit EGFR and VEGFR2 simultaneously. Therefore, in the present study, we designed computational screening methods to identify new potential compounds as dual tyrosine kinase inhibitor of EGFR and VEGFR2. The strategy includes three main stages. Firstly, we generated a validated pharmacophore model for EGFR and VEGFR2, and used it for initial screening of the ZINC database. Secondly, the hit compounds from the first stage were then screened again by molecular docking simulations against both targets using two docking tools. Finally, molecular dynamics simulations were performed for further validation of our screening result. 

## 2. Results and Discussion

### 2.1. Ligand-Based Pharmacophore Screening

**Figure 1:**
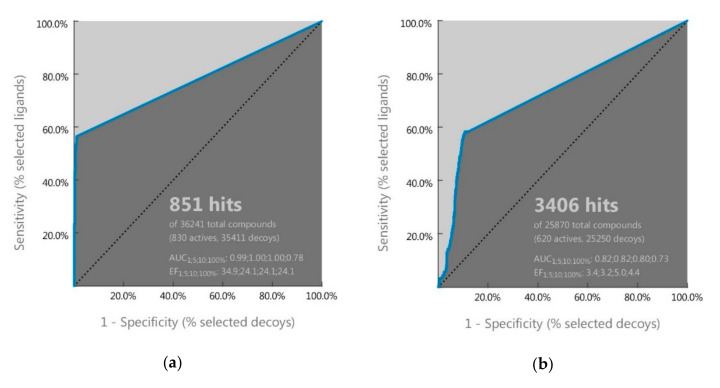
Receiver operating characteristic (ROC) curve validation of the selected pharmacophore model of (**a**) EGFR and (**b**) VEGFR2 using a set of active and decoy molecules.

In the present study, we built two ligand-based pharmacophore models, one for EGFR and the other for VEGR2 using LigandScout 4.3 on a set of known EGFR and VEGFR2 inhibitors. A total of 10 pharmacophore models were obtained for each target and the first model was selected as the best model based on its AUC value of receiver operating characteristic (ROC). As shown in Figure 1, both pharmacophore models had AUC100% of ROC ≥ 0.7, indicating that they could distinguish the active molecules from decoys and were categorized as valid.

**Figure 2:**
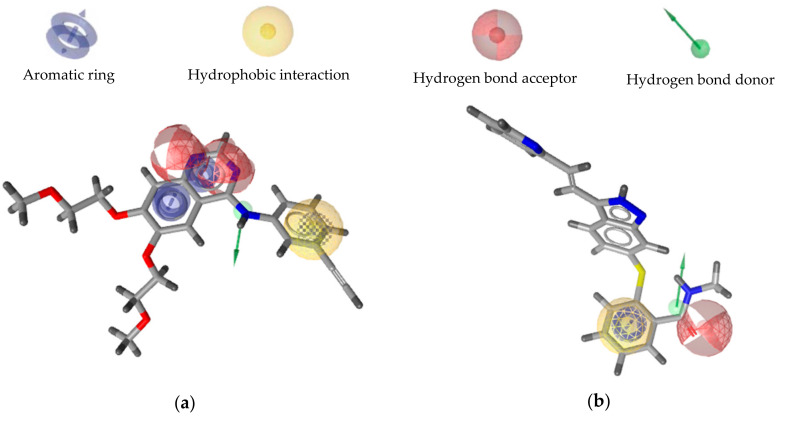
Overlay of erlotinib (**a**) and axitinib (**b**) on the selected pharmacophore model of EGFR and VEGFR2 inhibitor, respectively.

The selected pharmacophore model of EGFR consists of one hydrophobic group, three aromatic groups, two hydrogen bond acceptors, and one hydrogen bond donor, while the selected pharmacophore model of VEGFR2 consists of one hydrophobic group, one aromatic group, one hydrogen bond acceptor, and one hydrogen bond donor (Figure 2). 

The two pharmacophore models were then used to screen the ZINC database. Initially, the ZINC database was screened with pharmacophore models of EGFR, and 18,806 compounds were identified that could be mapped with the pharmacophore features. These compounds were then screened again with the pharmacophore model of VEGFR2, and it was obtained 6896 compounds that satisfied to our requirements for both models.

### 2.2. Molecular Docking Screening

In order to acquire dependable results, virtual screening was performed by two docking tools, DOCK6 and iGemdock. These docking tools were validated by redocking the native ligand to the active site of the target. The validation results showed that redocking of erlotinib into EGFR using DOCK6 and iGemdock gave a root-mean-square deviation (RMSD) value of 1.44 and 1.75 Å, respectively, while redocking of axitinib to VEGFR2 using DOCK6 and iGemdock gave RMSD value of 0.18 and 0.51 Å. A docking prediction is considered successful if the RMSD value is <2.0 Å for the best-scored conformation [18]. Therefore, DOCK6 and iGemdock have a good accuracy to put back erlotinib and axitinib into the binding pocket of EGFR and VEGFR2. The best-scored conformation of erlotinib after redocked in DOCK6 and iGemdock protocols were −64.26 and −107.61, respectively, whereas for axitinib in DOCK6 and iGemdock were −83.01 and −149.07, respectively. 

**Figure 3:**
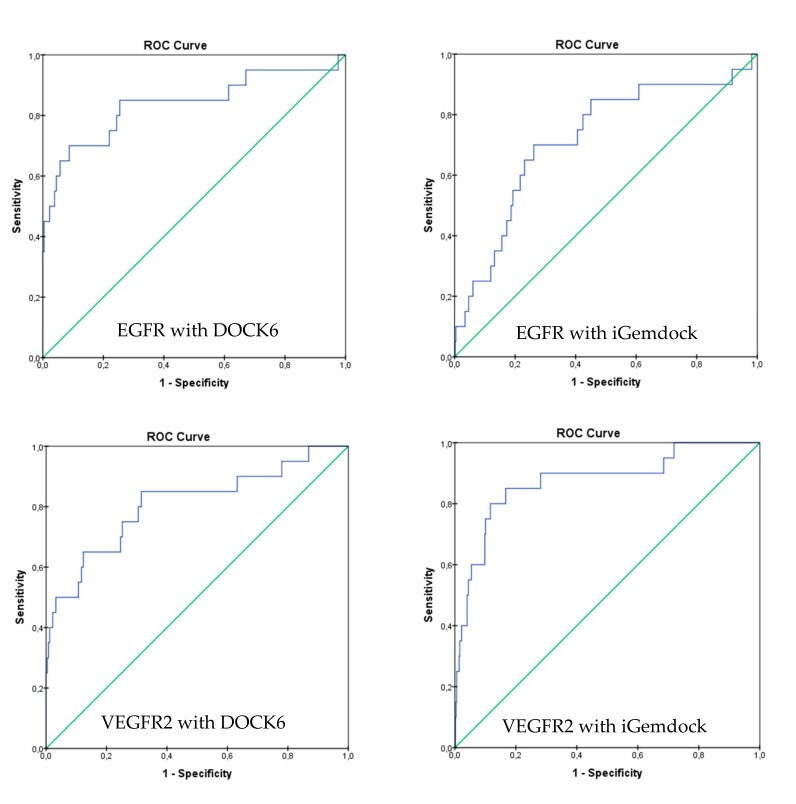
Receiver operating characteristic (ROC) curve validation of docking protocols using DOCK6 and iGemdock.

Moreover, analysis of the ROC curve was also performed to evaluate the ability of both docking protocols to discriminate between active and inactive compounds. As shown in Figure 3, DOCK6 and iGemdock have a steeper ROC curve than the random selection curve (diagonal curve). This indicates that these docking protocols are able to discriminate between active and inactive compounds. For verification, the AUC of the ROC curve for both docking methods were calculated. AUC of DOCK6 and iGemdock with EGFR as the target were 0.838 and 0.720, respectively, while toward VEGFR2, DOCK6 and iGemdock gave AUC of 0.809 and 0.874, respectively. AUC value of ≥0.7 could be considered that a method has acceptable discrimination [19]. Therefore, both of them could be applied for virtual screening. 

According to the best-scored conformation of erlotinib and axitinib, the 6896 hit compounds that satisfied the features of EGFR and VEGFR2 pharmacophores were then screened further by molecular docking. Firstly, the hit compounds were docked to EGFR and then continued to VEGFR2 using DOCK6. There were 67 hit compounds with more negative docking scores than those of erlotinib and axitinib. These compounds were then docked again to EGFR and then to VEGFR2 using the second docking tool, iGemdock, and there were 6 hits as potential dual inhibitor compounds.

### 2.3. Molecular Docking Analysis

**Figure 4:**
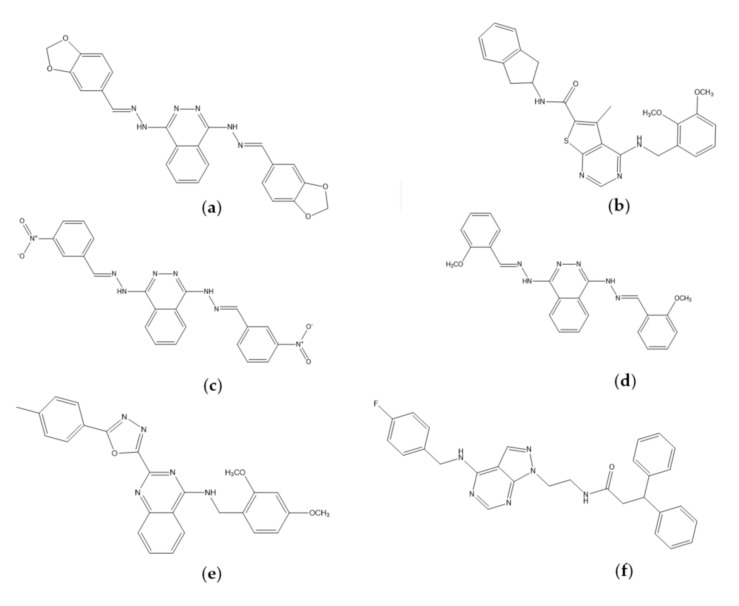
Chemical structure of (**a**) ZINC08398597, (**b**) ZINC12047553, (**c**) ZINC16525481, (**d**) ZINC17418102, (**e**) ZINC21942954, and (**f**) ZINC3848463.

We identified 6 compounds, i.e., ZINC08398597, ZINC12047553, ZINC16525481, ZINC17418102, ZINC21942954, and ZINC38484632 that meet all the filtering criteria. Interestingly, based on their chemical structure (Figure 4), some of them contain quinazoline- and phthalazine-based scaffold. Quinazoline ring has been commonly used in drug design studies for a dual inhibitor of EGFR and VEGFR2 [10,11], while the phthalazine ring is the isostere of the quinazoline ring [13]. These heterocyclic rings could serve as a scaffold that occupies the binding site for adenine of ATP in the hinge region [20]. 

In order to know the interaction profiles of our potential compounds with EGFR and VEGFR2, their best-scored conformation from docking simulations using DOCK6 were analyzed. We chose DOCK6 due to its accuracy better than iGemdock based on the RMSD value. According to docking studies, generally, our hit compounds were docked successfully into the binding site of EGFR and VEGFR2. As shown in Table 1, there were 4 compounds, i.e., ZINC08398597, ZINC12047553, ZINC21942954, and ZINC38484632 that formed a hydrogen bond with Met769 of EGFR. It must be noted that Met769 is recognized as a hinge region key residue in the binding of EGFR inhibitors with 4-anilinoquinazoline scaffolds, such as erlotinib [21]. 

In addition, some compounds also formed a hydrogen bond with Lys692, Lys721, Pro770, Cys773, Asp831. The existence of these residues in hydrogen bonds match with previously reported results by Yang et al. [22]. Especially on the Asp831, this residue is a part of the DFG sequence that plays a critical role in ATP binding. A study by Peng et al. demonstrated that the hydrogen bond between their ligands and Asp831 is important for enhancing the inhibitory activity [23]. Among the six compounds, only ZINC17418102 did not form hydrogen bonds with EGFR. However, it had 8 similar residues out of 10 EGFR residues in the erlotinib complex. Thereby, its affinity was predicted tends to be influenced by hydrophobic interaction. Furthermore, the molecular docking studies with VEGFR2 as the target (Table 2) showed that all compounds formed a hydrogen bond with Asp1046. This residue is equivalent to Asp831 at EGFR which is a part of the DFG motif. Four compounds, ZINC08398597, ZINC16525481, ZINC17418102, and ZINC38484632 also formed a hydrogen bond with Glu885 locating in the back pocket. Glu885 and Asp1046 are involved in the interaction of axitinib with VEGFR2, in addition to Glu917 and Cys919 which locate in the hinge region [24].

In contrast to other compounds, ZINC21942954 and ZINC38484632 also formed an additional hydrogen bond with Cys1045. In the 4AG8 structure, VEGFR2 is in an inactive form, which adopts DFG-out conformation [25]. This conformation provides an additional hydrophobic pocket for ligand interaction [24]. The six potential compounds have a moiety placed at this hydrophobic pocket. Therefore, we assumed that they will stabilize the DFG-out conformation and maintain VEGFR2 in its inactive form, and consequently inhibit the phosphorylation process. 

### 2.4. Molecular Dynamics Studies

#### 2.4.1. Stability Analysis

**Figure 5:**
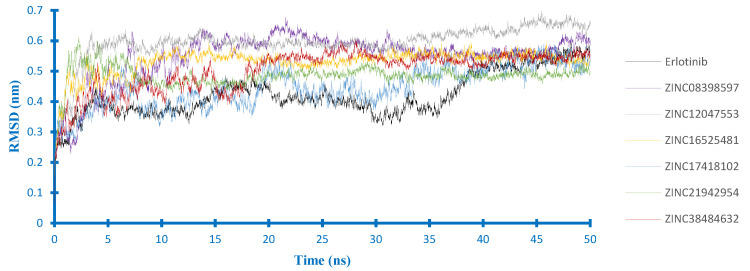
Backbone RMSD of the EGFR complexes.

In order to further evaluate the effect of solvent and flexibility of protein toward the binding mode of our candidate compounds, molecular dynamics (MD) simulations were carried out during 50 ns. Based on these simulations, the stability of the ligand-protein complexes was determined by the RMSD value. According to Figure 5, the EGFR backbone of each complex equilibrated at about 0.5 nm, except the complex of ZINC12047553 equilibrated at the higher value. The complex of erlotinib had a lower RMSD value at the start of the simulations. However, it still seemed to fluctuate at the end of the simulation. Furthermore, each complex reached the equilibration at different times. The complex of ZINC16525481 and ZINC12047553 equilibrated faster than others. Nevertheless, the ZINC12047553 complex had the highest RMSD (0.6 nm). These results suggested that the interaction of each compound had different effects on EGFR stability.

**Figure 6:**
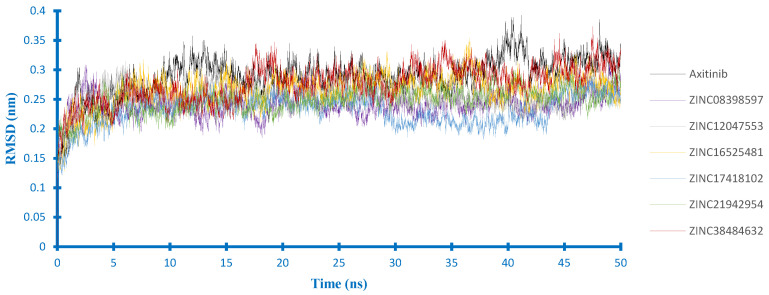
Backbone RMSD of the VEGFR2 complexes.

For their complex with VEGFR2 (Figure 6), axitinib, and all potential compounds influenced the stability of VEGFR2 at a similar level. However, RMSD of the VEGFR2 backbone in the axitinib-complex seemed higher than the others. Overall, no significant fluctuations were observed in the backbone of VEGFR2, which indicates that all protein structures were stable during 50 ns simulation.

**Figure 7:**
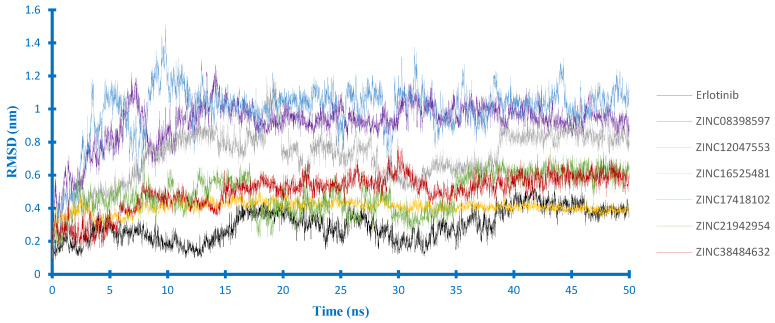
Ligand RMSD in the EGFR complexes.

**Figure 8:**
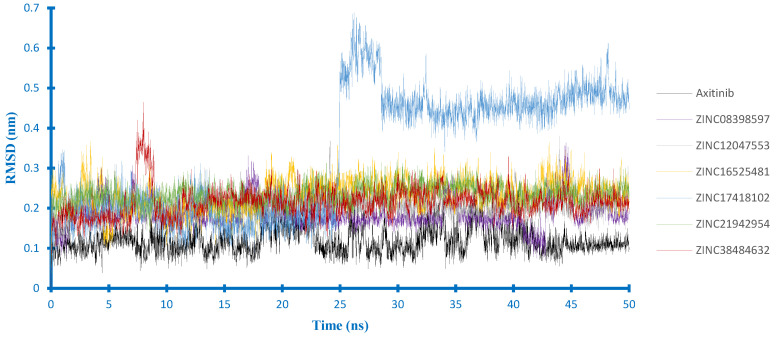
Ligand RMSD in the VEGFR2 complexes.

Furthermore, according to the ligand RMSD in the EGFR complex (Figure 7), there were three compounds (i.e., ZINC21942954, ZINC16525481and ZINC38484632) whose RMSD curves appeared to overlap at the lower value, indicating that their docking pose was more stable than others. Among them, ZINC16525481 had the average RMSD value close to erlotinib during the last 10 ns of MD simulations and quickly reached the equilibrium. In the VEGFR2 complex (Figure 8), five compounds had an average value of RMSD < 0.25 nm. The most stable ligand’s RMSD was found in ZINC08398597, with an average RMSD of 0.18 nm. Different from those of other compounds, ZINC17418102 showed a sharp increase of RMSD at around 25 ns. However, according to the visualization, this compound stays inside of the binding pocket, and it was observed that the high RMSD value at that time is related to the conformation change of ligand.

#### 2.4.2. Hydrogen Bond Analysis

In the present study, we have calculated the occupancy percentages of hydrogen bonds that more than 10% for all complexes during MD simulations. Based on the occupancy percentages, a hydrogen bond is considered to be stable when the occupancy is more than 50% [26]. The results showed that only two compounds, i.e., ZINC16525481, and ZINC38484632, which formed stable hydrogen bonds in their complex with EGFR and VEGFR2 as presented in Table 3. Interestingly, the stable hydrogen bonds were formed with the key residues of both targets. In the case of EGFR complexes, similar to erlotinib, ZINC16525481 showed nicely hydrogen bonds with Met769 through its N1 and N2 atoms of phthalazine ring as acceptors with occupancy 88% and 73.7%, respectively. Additionally, it was also found to form hydrogen with occupancy 74.3% at Gln767, whereas ZINC38484632 bound to Met769 with occupancy 92.1%; however, this interaction did not involve the atom of the heterocyclic ring of ZINC38484632. 

**Figure 9:**
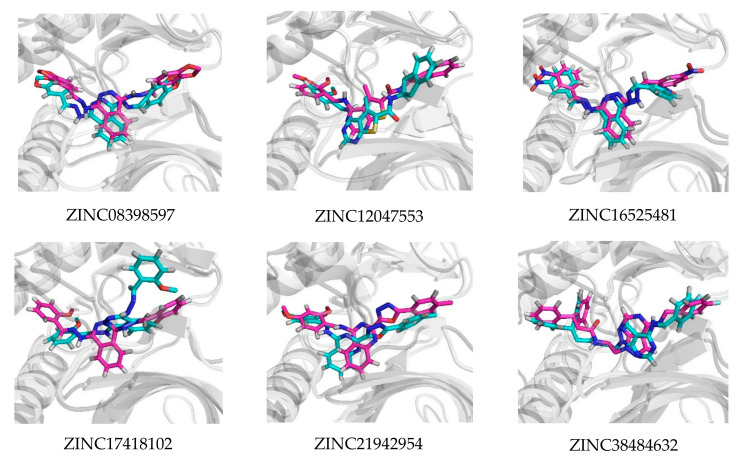
Superimposed ligand binding poses of the potential compounds at 0 ns (magenta) and 50 ns (cyan) in their complex with VEGFR2.

**Figure 10:**
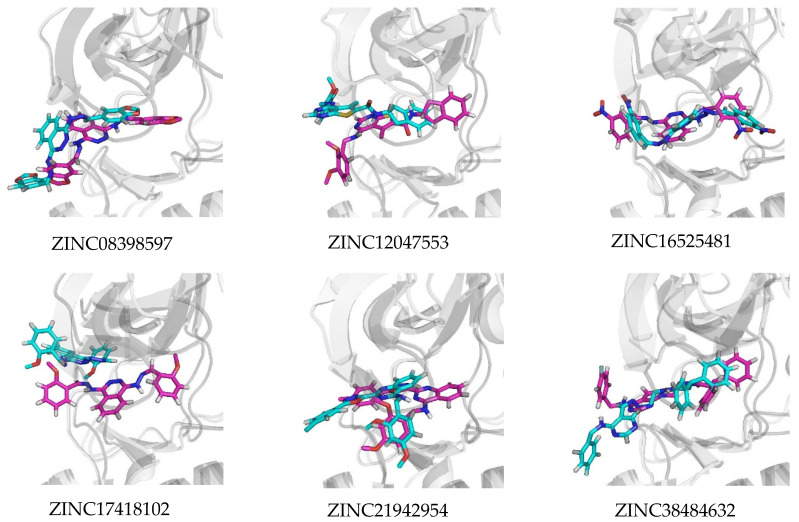
Superimposed ligand binding poses of the potential compounds at 0 ns (magenta) and 50 ns (cyan) in their complex with EGFR.

On the other hand, for their complex with VEGFR2, ZINC16525481 had two stable hydrogen bonds with Asp1046 (70.2%) and Cys919 (70.5%), while ZINC38484632 had a hydrogen bond at Asp1046 with occupancy 65%. In general, the results demonstrated that these potential compounds tend to show better binding interactions with VEGFR2 than EGFR. Moreover, Figure 9 showed that they also appear more stable to persist in the VEGFR2 binding pocket. This might be due to their number and occupancy percentages of hydrogen bonds in the VEGFR2 complex were higher than in the EGFR complex. In other words, the low number and percentage occupancy of hydrogen bonds are related to poor interaction stability. As shown in Figure 10, ZINC17418102, which has no hydrogen bond with occupancy of more than 10%, moved far away from its initial position in the binding pocket of EGFR. Similarly, the change in position of ZINC12047553 in the binding pocket was also observed. However, this compound still had one hydrogen bond with occupancy 25%, therefore its ligand stability appeared to be slightly better than ZINC17418102.

#### 2.4.3. MMPBSA Analysis 

**Figure 11:**
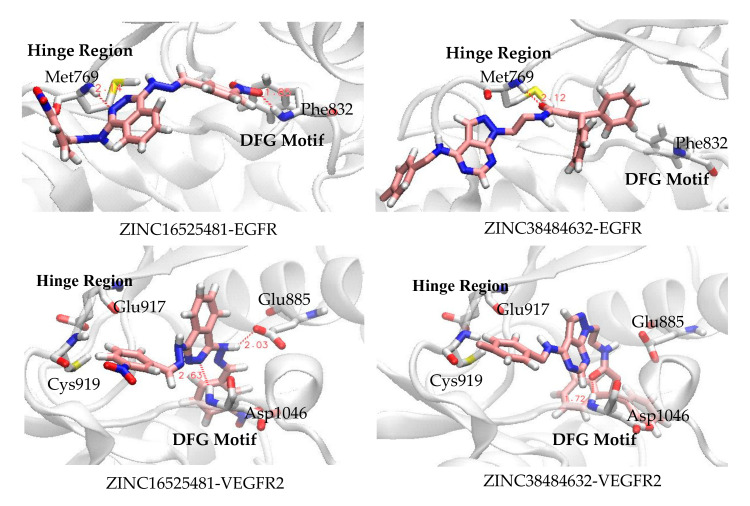
Binding mode of ZINC16525481 and ZINC38484632 to the ATP binding pocket of EGFR and VEGFR2 at 50 ns.

Based on the MD simulations, the binding free energy of the six potential compounds was calculated using The Molecular Mechanics Poisson–Boltzmann Surface Area (MMPBSA) method during their steady-state. As demonstrated in Table 4 and Table 5, energy decomposition to individual components showed that van der Waals, electrostatic, and SASA energy were favorable for ligand binding. In their interaction with EGFR, there were three compounds (ZINC08398597, ZINC16525481, ZINC38484632) with binding free energy < −100 kJ/mol like erlotinib. Among them, ZINC08398597 possessed the best binding energy, even better than erlotinib. Unfortunately, this compound exhibited a high ligand RMSD value of approximately 1 nm (10 Å) and it had been confirmed by visualization that it occupied an area slightly away from ATP binding pocket. Furthermore, ZINC38484632 and ZINC1652548 ranked at second and third place, respectively. However, among these three compounds, ZINC1652548 showed the van der Waals and electrostatic energy close to erlotinib, in correspondence with its interaction and RMSD profile. Meanwhile, the lowest free energy was observed in the ZINC17418102-EGFR complex. Based on its interaction profile, this compound did not form hydrogen bonds, either at the docking stage or during MD simulations. For their complex with VEGFR2, all six potential compounds got a better free binding energy than axitinib. This result indicated that they could bind to VEGFR2 with a good affinity. Compared to the other systems, the binding energy of ZINC08398597 was the best, while ZINC21942954 showed the lowest binding energy. Furthermore, based on the individual energy component, van der Waals energy of all the potential compounds was greater than axitinib, showing that the hydrophobic interaction is a dominant contributor for the binding of these potential compounds to VEGFR2. Thus, ZINC16525481 and ZINC38484632 were selected as a potential dual inhibitor for EGFR and VEGFR2 according to several parameters, such as their binding energy, RMSD profiles, hydrogen bond stability, and binding mode to the ATP binding pocket (Figure 11).

## 3. Materials and Methods 

### 3.1. Ligand-Based Pharmacophore Modeling

For each target, EGFR and VEGFR2, there were 26 known inhibitors as training set obtained from the literature. In order to generate pharmacophore models of EGFR, we used the same training set contain 26 active compounds that were compiled by Gupta et al. [14], while for VEGFR2, 26 active compounds with IC_50_ < 100 nM were selected from published literature by Zhang et al. [27]. Their structure was built and optimized using Hyperchem 8.0 [28] on the semiempirical method-Parametric Method 3 (PM3). These files were saved as .mol2 and loaded to LigandScout 4.3 [29], then generated their conformation using the default settings of iCon best option. Finally, LigandScout converted it into corresponding pharmacophore features. 

A total of 10 pharmacophore models were generated and the first model (model 1) was selected and validated for its performance to distinguish active compounds from decoys by screening a set of 830 known actives and 35,411 decoy compounds for target EGFR, and 620 known actives and 25,250 decoy compounds for target VEGFR2 obtained from DUD-E decoys database [30]. The database from DUD-E was converted in the .ldb format before screening by the “create screening database” menu of LigandScout. 

Initial screening was carried out using ZINCPharmer [31] for target EGFR. The previously obtained pharmacophore model of EGFR was submitted to ZINCPharmer, to search hits from the ZINC database of “purchasable compounds” consist of 22,723,923 compounds [32]. A maximum of 0.5 Å RMSD from sphere centers, 10 rotatable bonds cut-off, and the molecular weight no more than 500 Daltons were used as input parameters for ZINCPharmer. The database of hit compounds from the ZINCPharmer was downloaded and submitted to LigandScout in the .ldb format, and were then screened again using the pharmacophore model of VEGFR2. 

### 3.2. Molecular Docking

The hit compounds obtained from the pharmacophore modeling were used as input for further screening by docking simulations toward EGFR and VEGFR2 using DOCK6 [33] and iGemdock 2.1 [34]. The crystal structure of EGFR (PDB code: 1M17) and VEGFR2 (PDB code: 4AG8) were downloaded from the RSCB protein data bank (PDB). In the 1M17 structure, EGFR is in complex with a native ligand, erlotinib, while in the 4AG8, VEGFR2 is in complex with axitinib. Swiss–Model was used for adding missing residues of the proteins [35].

For docking using DOCK6, the proteins without water molecules were separated from their native ligands using Chimera [36]. The proteins and native ligands were prepared by the dock prep tool of Chimera. This stage includes the addition of hydrogen atoms and standard charges. Specifically for the proteins, the molecular surface was generated with the DMS tool, as provided by DOCK6 software, with a default probe radius of 1.4 Å. The DMS output with default settings of Sphegen was applied to generate sphere, while the sphere selector tool and an 8 Å radius about the native ligands was used to define the active site. Moreover, 5 Å extra margin in all six directions was applied to support the box around the active site. At the last step, flexible ligand docking was used for virtual screening with default input parameters according to the DOCK6 tutorial.

For docking with iGemdock, only water molecules were removed from protein-ligand complexes. The complexes were loaded to iGemdock and their binding sites were identified at a distance 5 Å of the bounded ligands. The stable docking module was used for virtual screening with the default parameters such as population size = 300, generations = 80, number of solutions = 10. 

The accuracy of both docking protocols was validated by redocking native ligand into the binding pocket of the protein and its RMSD value was calculated. These docking protocols were also validated for their performance in distinguishing between active and decoy compounds by analyzing AUC of ROC after screening the DUD-E database of EGFR and VEGFR2.

### 3.3. Molecular Dynamics Simulations

The topologies of native ligands and candidate compounds were generated using Automated Topology Builder (ATB) 3.0 [37]. All MD simulations were performed using the GROMACS 2016.3 package [38] under simple point charge (SPC) water mode and GROMOS96 54A7 force field. The simulation of system was run under the periodic boundary condition with a dodecahedron periodic box, which was also solvated by SPC water molecules. The surface of the enzyme was covered with a water shell of 1.0 nm. The balance of system charge was achieved by adding sodium and chloride ions. The relaxation of complex system was conducted by energy minimization under 1000 kcal/mol/nm by using the steepest descent method. A 250 ps NVT (constant number of particles, volume, and temperature) ensemble was then performed to stabilize the system at 310 K and subsequently, 250 ps NPT (constant number of particles, pressure, and temperature) equilibration stabilized the system pressure by using coupling reference pressure of 1 bar. The last step, MD simulation, was conducted for 50 ns with a time step of 2 fs, and the corresponding coordinates were stored every 2 ps. Particle Mesh Ewald (PME) was applied to calculate the long-range electrostatics during the simulation. In the present work, to calculate the binding energy of each complex from the MD trajectories, we used the g_mmpbsa tool and its protocols as reported by Kumar et al. [39]. The calculation of binding energies was based on the extracted 20 snapshots from the last 5 ns trajectory of 50 ns simulation results.

## 4. Conclusions

In the present study, we developed a computational method combining ligand-based pharmacophore filtering and molecular docking for identifying the potential compounds that can inhibit tyrosine kinase of EGFR and VEGFR2, simultaneously. The results showed that there were six compounds mapped well onto the features of our selected pharmacophore models and had a better docking score than erlotinib and axitinib as the reference drugs. Additionally, the binding mode analysis revealed that they also had a similar interaction with the reference drugs. However, after careful analysis of their binding mode stability during molecular dynamics simulations, it was found that there were two compounds, ZINC16525481 and ZINC38484632 retained the stable hydrogen bonds (occupancy > 50%) with the essential residues of both targets, with a good binding free energy, indicating that these compounds were able to steadily anchor to the binding pocket of EGFR and VEGFR2 to exert an inhibitory effect. Thus, the results suggested that ZINC16525481 and ZINC38484632 could be considered as a potential dual inhibitor for EGFR and VEGFR2 for further study. The study of biological activities of two compounds is currently underway and will be reported elsewhere.

## Figures and Tables

**Table 1:** EGFR-ligand interactions recorded during docking.

Ligand	DOCK6 Score	iGemdock Score	H-Bond	Hydrophobic interaction
Erlotinib	−64.26	−107.61	Met769	Leu694, Ala719, Lys721, Leu764, Thr766, Leu768, Gly772, Leu820, Asp831
ZINC08398597	−68.76	−129.93	Met769	Leu694, Ala719, Val702, Leu768, Pro770, Gly772, Asp776, Tyr777, Leu820
ZINC12047553	−72.76	−125.93	Met769	Leu694, Ala719, Val702, Lys721, Met742, Leu764, Pro770, Gly772, Asp776, Leu820
ZINC16525481	−73.03	−128.81	Lys692, Lys721, Asp831	Leu694, Ala719, Lys721, Met742, Pro770, Gly772, Cys773, Leu820
ZINC17418102	−65.89	−122.16	-	Lys692, Leu694, Ala719, Val702, Lys704, Lys721, Leu764, Thr766, Pro770, Gly772, Leu820, Asp831
ZINC21942954	−72.93	−117.69	Met769, Cys773, Asp831	Leu694, Gly695, Lys721, Leu768, Pro770, Gly772, Leu820
ZINC38484632	−72.97	−111.43	Met769, Pro770	Lys692, Leu694, Ala719, Val702, Lys721, Met742, Thr766, Leu768, Gly772, Leu820

**Table 2:** VEGFR2-ligand interactions recorded during docking.

Ligand	DOCK6 Score	iGemdock Score	H-Bond	Hydrophobic interaction
Axitinib	−83.00	−149.07	Glu885, Glu917, Cys919, Asp1046	Leu840, Ala866, Lys868, Val916, Phe918, Gly992, Leu1035, Phe1047
ZINC08398597	−89.65	−156.25	Glu885, Asp1046	Leu840, Val848, Ala866, Val867, Lys868, Leu889, Val914, Val916, Gly992, Leu1019, His1026, Leu1035, Phe1047
ZINC12047553	−85.20	−168.46	Asp1046	Leu840, Val848, Ala866, Lys868, Glu885, Val899, Val914, Val916, Phe918, Cys919, Gly992, His1026, Leu1035, Ile1044, Cys1045, Phe1047
ZINC16525481	−94.84	−162.89	Glu885, Asp1046	Leu840, Val848, Ala866, Val867, Lys868, Leu889, Val914, Val916, Phe918, His1026, Leu1035, Cys1045, Phe1047
ZINC17418102	−89.39	−155.33	Glu885, Asp1046	Leu840, Val848, Ala866, Val867, Lys868, Leu889, Val914, Val916, Glu917, Phe918, His1026, Leu1035, Ile1044, Phe1047
ZINC21942954	−84.25	−152.80	Cys1045, Asp1046	Leu840, Lys868, Leu889, Val899, Val914, Val916, Phe918, Cys919, Leu1035, Ile1044, Phe1047
ZINC38484632	−85.05	−151.26	Glu885, Cys1045, Asp1046	Leu840, Val848, Ala866, Lys868, Leu889, Ile892, Val916, Cys1024, Leu1019, Ile1025, His1026, Leu1035, Phe1047

**Table 3:** Occupancy of hydrogen bond during 50 ns simulations.

Ligand	Target			
	EGFR		VEGFR2	
	Donor-Acceptor	Occupancy (%)	Donor-Acceptor	Occupancy (%)
Erlotinib	Met769 (H)---(N2)	28.1	-	-
Axitinib	-	-	(H12)---Glu917 (O)	76.5
			(H1)---Glu885 (OE2)	30.3
			(H1)---Glu885 (OE1)	27.7
			Asp1046 (H)---(O81)	69.5
			Cys919 (H)---(N14)	88.8
ZINC08398597	Cys773 (H)---(N1)	21.1	(H12)---Glu885 (OE2)	41.5
	Cys773 (H)---(N2)	13.4	(H12)---Glu885 (OE1)	34.5
	Met769 (H)---(O4)	10.2	Asp1046 (H)---(N6)	32.5
			Asp1046 (H)---(N1)	45.6
ZINC12047553	Met769 (H)---(O1)	25.1	(H15)---Asp1046 (O)	11.5
ZINC16525481	(H5)---Gln767 (O)	74.3	(H11)---Glu885 (OE2)	17.6
	Phe832 (H)---(O)	45.4	(H11)---Glu885 (OE1)	17.4
	Met769 (H)---(N1)	88	Asp1046 (H)---(N1)	70.2
	Met769 (H)---(N2)	73.7	Asp1046 (H)---(N2)	47.7
			Cys919 (H)---(O)	70.5
ZINC17418102	-	-	Asp1046(H)---(N6)	41.3
ZINC21942954	Cys773 (H)---(O3)	48.7	(H12)---Glu885 (OE2)	31.5
			(H12)---Glu885 (OE1)	27.4
			Asp1046 (H)---(N4)	37.4
			Asp1046 (H)---(N5)	24.3
ZINC38484632	(H16)---Pro770 (O)	10.5	(H9)---Glu885 (OE2)	16.8
	Cys773 (H)---(N4)	16.8	(H9)---Glu885 (OE1)	19.8
	Met769 (H)---(O1)	92.1	Asp1046 (H)---(O1)	65.3

**Table 4:** The predicted binding free energy and the individual energy components (kJ/mol) ligand-EGFR complexes.

Ligands	Van der Waals Energy (Δ*E*_vdW_)	Electrostatic Energy (Δ*E*_elec_)	Polar Solvation Energy (Δ*G*_polar_)	SASA Energy (Δ*G*_nonpolar_)	Binding Energy (Δ*G*_bind_)
Erlotinib	−232.64 ± 13.43	−46.89 ± 10.03	176.32 ± 17.18	−24.18 ± 0.92	−127.38 ± 17.58
ZINC08398597	−220.76 ± 10.87	−24.78 ± 7.60	114.24 ± 14.70	−23.37 ± 1.72	−154.67 ± 13.85
ZINC12047553	−183.44 ± 16.81	−23.33 ± 7.92	131.99 ± 29.16	−19.71 ± 1.58	−94.49 ± 19.15
ZINC16525481	−223.17 ± 12.92	−43.01 ± 12.74	182.64 ± 14.30	−22.30 ± 0.99	−105.84 ± 17.08
ZINC17418102	−131.71 ± 12.28	−16.65 ± 10.30	85.25 ± 20.67	−15.09 ± 1.43	−78.21 ± 11.57
ZINC21942954	−200.15 ± 14.24	−16.94 ± 12.14	143.44 ± 29.17	−19.55 ± 1.2	−93.20 ± 18.56
ZINC38484632	−179.60 ± 11.35	−33.06 ± 8.88	118.60 ± 20.60	−21.25 ± 1.24	−115.31 ± 19.27

**Table 5:** The predicted binding free energy and the individual energy components (kJ/mol) ligand-VEGFR2 complexes.

Ligands	Van der Waals Energy (Δ*E*_vdW_)	Electrostatic Energy (Δ*E*_elec_)	Polar Solvation Energy (Δ*G*_polar_)	SASA Energy (Δ*G*_nonpolar_)	Binding Energy (Δ*G*_bind_)
Axitinib	−202.38 ± 11.43	−75.49 ± 10.19	163.49 ± 10.45	−20.69 ± 0.63	−135.08 ± 7.94
ZINC08398597	−267.39 ± 13.12	−32.26 ± 8.51	154.24 ± 12.60	−25.56 ± 0.76	−170.96 ± 12.17
ZINC12047553	−258.36 ± 10.04	−24.89 ± 8.73	163.99 ± 21.33	−26.22 ± 1.16	−145.48 ± 23.74
ZINC16525481	−272.50 ± 11.94	−29.68 ± 8.44	184.00 ± 15.54	−26.25 ± 1.03	−144.43 ± 12.32
ZINC17418102	−252.86 ± 10.25	−28.79 ± 6.46	166.57 ± 12.21	−24.63 ± 1.13	−139.71 ± 14.81
ZINC21942954	−268.09 ± 9.85	−42.85 ± 6.59	199.97 ± 25.24	−26.72 ± 0.73	−137.69 ± 25.23
ZINC38484632	−260.64 ± 9.06	−25.80 ± 7.40	167.18 ± 16.01	−27.72 ± 1.15	−146.97 ± 12.43
